# Climate change and tuberculosis: an analytical framework

**DOI:** 10.1016/S2213-2600(25)00329-7

**Published:** 2025-10-30

**Authors:** Matthew J Saunders, Delia Boccia, Palwasha Y Khan, Lara Goscé, Antonio Gasparrini, Rebecca A Clark, Julia M Pescarini, Salome Charalambous, Lelisa Fekadu, Fernanda Dockhorn da Costa Johansen, Irina Vasilyeva, Gopalan Narendran, Tao Li, Norbert Ndjeka, Richard G White, Rein M G J Houben, Matteo Zignol, Nebiat Gebreselassie, Christopher Finn McQuaid

**Affiliations:** Faculty of Public Health and Policy, London School of Hygiene and Tropical Medicine, London, UK; Institute for Infection and Immunity, City St George’s, University of London, London, UK; Faculty of Epidemiology and Population Health, London School of Hygiene and Tropical Medicine, London, UK; Department of Clinical Research, London School of Hygiene and Tropical Medicine, London, UK; TB Modelling Group, TB Centre, and Centre for Mathematical Modelling of Infectious Diseases, Department of Infectious Disease Epidemiology, London School of Hygiene and Tropical Medicine, London, UK; Environment and Health Modelling Laboratory, Department of Public Health, Environments and Society, London School of Hygiene and Tropical Medicine, London, UK; TB Modelling Group, TB Centre, and Centre for Mathematical Modelling of Infectious Diseases, Department of Infectious Disease Epidemiology, London School of Hygiene and Tropical Medicine, London, UK; Faculty of Epidemiology and Population Health, London School of Hygiene and Tropical Medicine, London, UK; Centro de Integração de Dados e Conhecimentos para Saúde (Cidacs), Instituto Gonçalo Moniz, Fundação Oswaldo Cruz, Salvador, Brazil; Aurum Institute, Johannesburg, South Africa; Ministry of Health, Addis Ababa, Ethiopia; Ministry of Health, Brasilia, Brazil; Ministry of Health, Moscow, Russia; National Institute for Research in Tuberculosis, Chennai, India; Chinese Center for Disease Control and Prevention, Beijing, China; National Department of Health, Pretoria, South Africa; TB Modelling Group, TB Centre, and Centre for Mathematical Modelling of Infectious Diseases, Department of Infectious Disease Epidemiology, London School of Hygiene and Tropical Medicine, London, UK; Global Tuberculosis Programme, World Health Organization, Geneva, Switzerland; TB Modelling Group, TB Centre, and Centre for Mathematical Modelling of Infectious Diseases, Department of Infectious Disease Epidemiology, London School of Hygiene and Tropical Medicine, London, UK

## Abstract

Climate change is likely to exacerbate a range of determinants that drive tuberculosis, the world’s leading cause of death from a single infectious agent. However, tuberculosis is often neglected in wider climate health discussions. Commissioned by WHO, we developed an analytical framework outlining potential causal relationships between climate change and tuberculosis. We drew on existing knowledge of tuberculosis determinants, identified determinants likely to be sensitive to the effects of climate change, and conceptualised the mechanistic pathways through which these effects might occur. We collated evidence for these pathways, but found no studies directly linking climate change and tuberculosis, warranting research to build evidence for action. Nevertheless, the available indirect evidence supports the existence of plausible causal links between climate change and tuberculosis. This evidence highlights the need to consider tuberculosis as a climate-sensitive disease, and include tuberculosis in climate risk adaptation and mitigation programmes, and climate-resilient funding and response mechanisms. Only through urgent research and comprehensive action can we address this overlooked intersection and ensure that climate change does not become a barrier to ending the global tuberculosis epidemic.

## Introduction

The health effects of climate change operate through complex and interconnected pathways, as outlined in the WHO Framework on Climate Change and Health, and further characterised in the Sixth Assessment Report of the Intergovernmental Panel on Climate Change (IPCC) and *Lancet* Countdown on climate change and health.^1–3^ Briefly, the WHO Framework postulates that climate-related hazards (eg, extreme weather events or sea level rise) interact with vulnerabilities (eg, gender or comorbidities), and exposures (eg, food and health systems) leading to direct and indirect health effects. These include injuries and mortality; increases in zoonotic diseases, foodborne, waterborne, vector-borne, and non-communicable diseases; and mental ill health.^4^ Climate change is also already affecting determinants of health by driving poverty, causing migration and displacement, worsening food and water insecurity, and disrupting access to health care and support systems.^1–3^ Importantly, many of the effects of climate change are cascading and compounding, and disproportionately affect populations in low-income and middle-income countries, particularly the most vulnerable, where resilience and ability to adapt are lower.^1–3^

These climate-sensitive determinants of health significantly overlap with key determinants of the global tuberculosis epidemic.^5^ Despite progress, tuberculosis continues to rank among the world’s leading causes of death.^6^ In 2023 alone, an estimated 10·8 million people fell ill from tuberculosis, and 1·25 million lost their lives to the disease.^6^ Determinants that heighten exposure to *Mycobacterium tuberculosis*, the causative agent of tuberculosis, such as overcrowding and poor living conditions (including in specific contexts such as prisons or congregate settings), increase the risk of transmission and subsequent infection (hereafter referred to as tuberculosis infection). Meanwhile, determinants more specifically linked to health, which principally impair the immune system, such as undernutrition, HIV, alcohol use disorders, smoking, and diabetes, increase the risk of progressing to symptomatic or infectious disease (referred to here as tuberculosis disease, to distinguish it from tuberculosis infection)^6^ and might worsen health outcomes. Many of these determinants can further exacerbate the known financial and psychosocial burden associated with tuberculosis, driving tuberculosis-affected households further into poverty.

**Figure 1:**
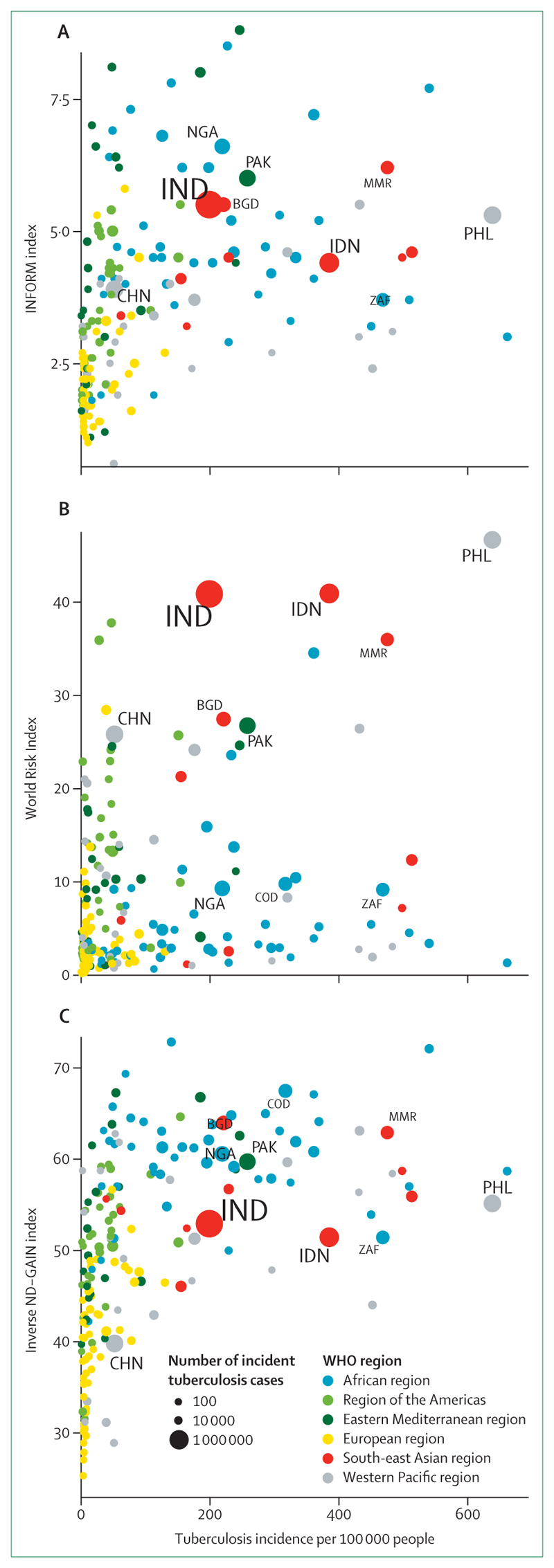
Comparison of tuberculosis burden and vulnerability to climate change across 215 countries and territories A higher index value represents a poorer performance, where indices include INFORM measuring the risk of humanitarian crises that could require international assistance (A); World Risk Index assessing the risk of humanitarian disaster caused by extreme natural events and the negative effects of climate change (B); and ND-GAIN combining both readiness and vulnerability to climate change (C). Colours indicate WHO regions. Dot sizes provide relative context of number of incident tuberculosis cases. ISO3 codes indicate the top ten tuberculosis burden countries by number of incident tuberculosis cases. BGD=Bangladesh. CHN=China. COD=Congo. IDN=Indonesia. IND=India. MMR=Myanmar. NGA=Nigeria. PAK=Pakistan. PHL=Philippines. ZAF=South Africa.

Notably, many countries with a high tuberculosis burden, including India, Indonesia, and the Philippines,^6^ are also highly vulnerable to the effects of climate change, as measured by several recognised indices (figure 1).^7–9^ Positioning tuberculosis in the context of climate change has, however, been overlooked due to insufficient research and advocacy. A scoping review suggested that climate change increases tuberculosis infection and disease risk, particularly among vulnerable populations,^10^ whereas a second review described the potential effects of climate change on several of the tuberculosis determinants described above.^11^ Despite these findings, the available evidence has not been systematically mapped against a comprehensive framework describing the potential pathways linking climate change and tuberculosis. Although some frameworks have been proposed,^12,13^ further work is needed to establish a global, consensus-based framework on the basis of evidence. The effects of climate change on tuberculosis are often overlooked in wider climate and health discussions,^14,15^ and no coherent strategy exists to help countries mitigate these impacts. Establishing a global framework is therefore urgently needed to guide effective policy and action.

In response to this gap, WHO Global Tuberculosis Programme commissioned the creation of an analytical framework outlining potential causal relationships between climate change and the tuberculosis epidemic, and research gaps to facilitate evidence-building for action. Here, we describe the creation of this framework and its comparison to existing evidence, identify research domains in the area of climate change and tuberculosis critically lacking in evidence, and suggest examples of entry points for intervention.

## Methods

Development of this framework followed an iterative review process. We first convened an internal working group of tuberculosis and climate researchers to develop key questions informing the creation of a preliminary analytical framework, drawing on existing literature, systematic reviews, and WHO publications on tuberculosis determinants, and wider discussions with key informants and experts (ie, the authors of this Position Paper). WHO then convened a multistakeholder consultation on the impact of climate change on the tuberculosis response, where attendees reviewed the analytical framework, supporting evidence, and research gaps. The consultation included 40 participants, including WHO, researchers, National TB Programme and Ministry of Health staff, funders, and civil society. Particpants were from Egypt, Saudi Arabia, South Africa, the UK, Zambia, France, Canada, Brazil, Ethiopia, the USA, Switzerland, China, India, Pakistan, Russia, Iran, Burkina Faso, and Australia. The framework was updated and refined following this meeting.

Our internal working group first identified the principal social and health determinants of tuberculosis and conceptualised how these determinants affect different aspects of the tuberculosis epidemic and response. We then selected which of these social and health determinants were likely to be sensitive to the effects of climate change, based on previous reviews,^10,11^ and hypothesised the causal mechanisms through which these effects might occur. This process included identifying relevant climate factors (eg, changing rainfall patterns or land degradation), the pathway of influence (eg, socioeconomic changes or migration), and how these factors might affect tuberculosis (eg, increasing transmission or worsening health outcomes).

After developing and visualising these hypothesised causal relationships in a preliminary analytical framework, we undertook a narrative literature review. This gathered and synthesised the best available evidence to support, reject, or refine our hypotheses, focusing on three examples of critical pathways: (1) migration and displacement; (2) food and water insecurity; and (3) health system disruptions. These example pathways, which are described further below and are termed climate–health links from hereon, were selected based upon: (1) the plausibility of their relationship to climate change, ie, to what extent a particular pathway was hypothesised to be sensitive to the effects of climate change; (2) their plausibility and importance for tuberculosis, informed by their relevance to key WHO End TB Strategy16 indicators and social and health determinants of tuberculosis emphasised by WHO; and (3) the likely availability of existing data and other types of evidence, and their amenability for future analysis.

Although each climate–health link could potentially influence multiple consequences for tuberculosis, including tuberculosis infection, tuberculosis disease, and tuberculosis outcomes, to simplify the process we tested the framework by focusing our literature review on a single tuberculosis-related consequence for each climate–health link. For example, we reviewed the effect of health system disruptions on tuberculosis outcomes, but did not conduct a comprehensive review of other potential consequences associated with the same climate–health link, such as an increase in transmission due to delayed diagnoses, or higher tuberculosis disease rates resulting from reduced preventive treatment and BCG vaccination coverage, as well as potentially impaired safety and efficacy. As a result, the evidence identified in our Position Paper is expected to represent a conservative perspective of the overall consequences for tuberculosis of each climate–health link. We undertook six searches of the MEDLINE database: three investigating the effect of climate change on each climate–health link; and three investigating the effect of each climate–health link on the consequence for tuberculosis hypothesised as the primary pathway for that link.

### Analytical framework

**Figure 2:**
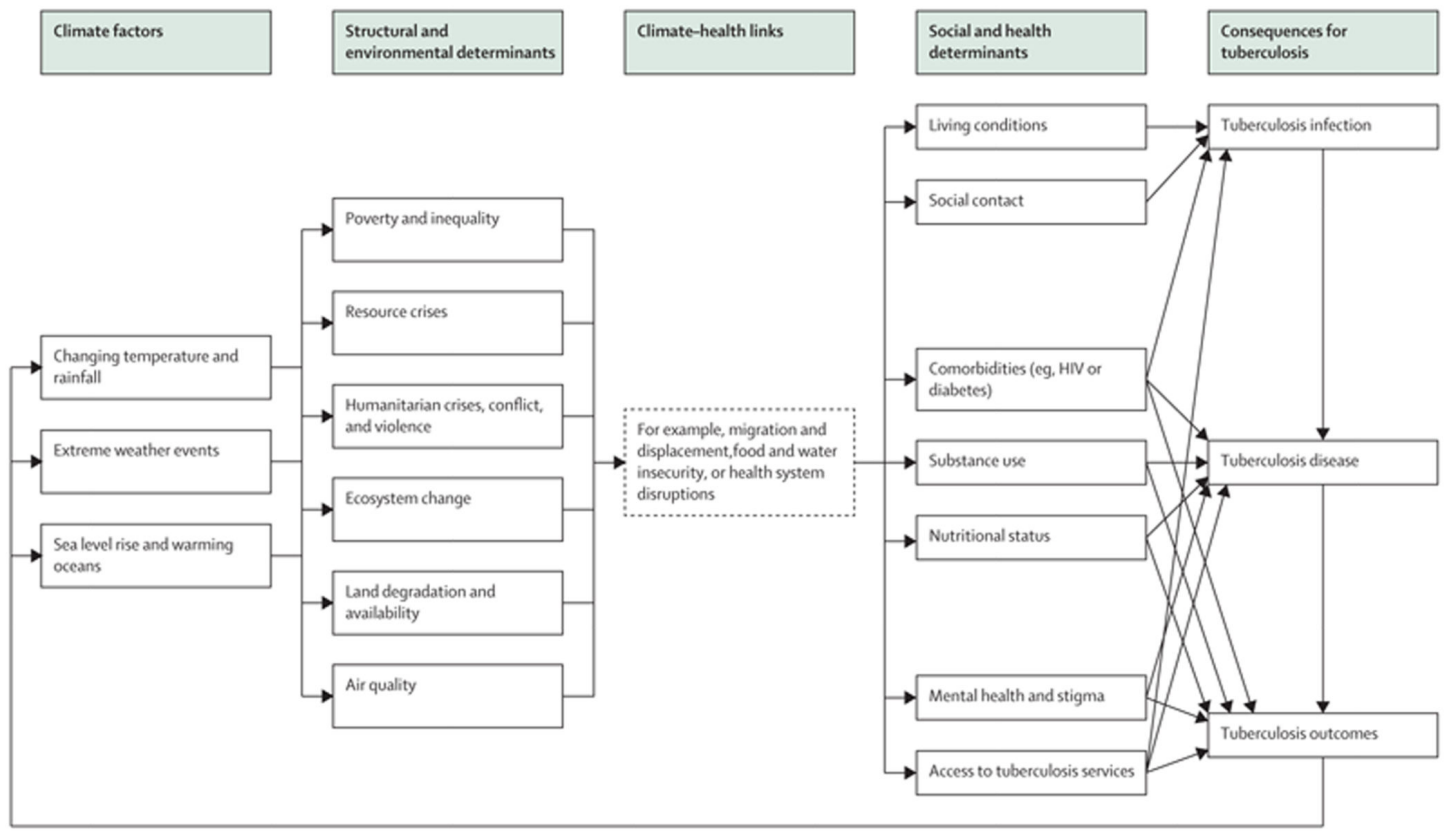
Analytical framework linking climate change to consequences for tuberculosis via a range of climate–health links

The resulting framework is presented in figure 2. At the highest level, elements are captured describing changing climate factors. These cover key examples, which are not exhaustive, such as changing temperatures and rainfall (leading to, eg, increased duration and frequency of droughts or extreme heat), rising sea levels and warming of oceans, and extreme weather events (such as flooding, storms, fires, droughts, and extreme heat). Factors are incorporated at a range of timescales, including whether the effects are expected to be visible in the near or longer-term future, and how long a given effect might last. For example, extreme weather events have already been widely recorded worldwide, and generally result in an immediate but (per event) shorter-term effect. By contrast, sea level rises might currently be a more distant prospect, but one that will likely have a longer-term effect.

As a direct result of these climate factors, a variety of structural and environmental determinants of tuberculosis are potentially affected. These cover examples including levels of poverty and inequality (both within and between countries), resource crises (such as fuel, housing, materials, and other resources), humanitarian crises, conflict, and violence over resource competition (and related impacts such as increased incarceration), ecosystem change (including changes in seasonality, and in the living and non-living components of ecosystems), land degradation and availability (including changes to vegetation and available farmland), and air quality.

Changing structural and environmental determinants are then linked to health via a series of climate–health links. Three prioritised examples of links are outlined in the sections below and in table 1: migration and displacement, food and water insecurity, and health system disruption. Each link completes a direct causal pathway through which changes in structural and environmental determinants drive changes in exposure to social and health determinants.

Social and health determinants cover examples such as living conditions and housing (affecting factors such as overcrowding and ventilation); changing social contact as individuals move within and between countries; and changes in prevalence and management of comorbidities including (but not limited to) HIV and diabetes, changes in nutritional status, mental health and stigma, and provision of and access to tuberculosis prevention and care services.

Lastly, social and health determinants are directly linked to tuberculosis consequences, where they affect (1) the likelihood of exposure to and susceptibility to tuberculosis infection; (2) the risk of progression to tuberculosis disease; and (3) the extent of vulnerability to tuberculosis outcomes (including short-term and long-term morbidity, disabilities, mortality, acquisition of drug-resistance, and psychological and financial consequences).

These elements can impact progress towards the goals and targets of the WHO End TB strategy.^16^ Increased tuberculosis exposure and susceptibility might drive higher transmission, leading to greater infection rates, disease burden, and associated health and social costs. Similarly, tuberculosis disease progression influences health outcomes, onward transmission and incidence, and tuberculosis-related socioeconomic consequences. Moreover, worsening inequalities can exacerbate health disparities, including mortality and tuberculosis-related costs.

Tuberculosis care and prevention programmes might also contribute to climate change, creating a feedback loop within the framework. The health-care sector more generally is estimated to account for approximately 5% of global emissions,^17^ and when specifically considering tuberculosis, there are multiple sources of emissions across the tuberculosis care cascade.^18,19^ These emission sources include energy and material inputs required for tuberculosis diagnosis and treatment, transportation emissions from patient visits and sample transport, and the medical and biological waste generated as part of providing care.

Due to the interconnectedness of potential pathways, many of which are cyclic or mutually reinforcing, the framework is not intended to be exhaustive. Instead, the framework seeks to capture the pathways with significant implications for the tuberculosis epidemic and response, and those that are amenable to analysis and actionable from a policy perspective. We did not explore the bidirectionality of some relationships (eg, the effect of tuberculosis disease on nutritional status), or highlight interactions between different determinants (such as between resource crises, poverty, and inequality). As part of this work, the pathways for the three climate–health links described above were reviewed in detail (table 1).

Importantly, these pathways often create compounding vulnerabilities. For example, displaced populations frequently have reduced access to adequate nutrition and healthcare; those living in poverty who are experiencing food insecurity often concurrently have limited access to health care and might have higher displacement risks; and when extreme weather events cause disruptions to health systems, they typically also cause displacement and disruptions to food security.

### Migration and displacement

#### Evidence on climate change, migration, and displacement

Climate change and migration and displacement has an extensive history of research,^3^ with multiple existent frameworks20,21 and a wealth of examples of how climate change might drive population movement both between and, more commonly, within countries.^22–26^ In 2023, approximately 20·3 million people were internally displaced as a result of weather-related hazards, and by 2050 that number could increase to 216 million due to slow-onset climate change impacts.^27^ Several reviews cover circumstances encompassing natural hazards and socioeconomic changes,^28–31^ including reviews comparing different climate migration models.^32–34^ Models projecting future migration due to climate change are also numerous. Multiple examples focus on sea level rise and flooding;^35–38^ although some consider meteorological,^39–43^ macroeconomic,^44,45^ and agricultural changes,^46^ and frameworks considering multiple migration drivers exist.^47,48^ Given this evidence, the IPCC states with high confidence that extreme weather events and variability act as direct drivers of involuntary migration and displacement, and as indirect drivers through deteriorating economic conditions and livelihoods.^3^ However, patterns of migration due to climate change are likely to be highly context specific and are difficult to project because of the multicausal nature of migration, migration policies, and the scale and nature of any future adaptations. Nevertheless, it is striking that the IPCC states that under all global warming levels there are areas of the world that will become unsafe or uninhabitable, many of which are currently densely populated and have a high tuberculosis burden.^3^

#### Evidence on migration, displacement, and tuberculosis infection

There is also a large body of evidence linking migration (particularly forced migration and displacement) to higher tuberculosis risk. Although no routine estimates of burden are published, reviews suggest a multifold increased risk of tuberculosis for people who are displaced, varying substantially by migrant type and context.^49,50^ The literature focuses principally on the additional tuberculosis disease burden and poor treatment outcomes (partly because of reduced access to care) experienced by people who are displaced or those in crisis situations,^49–53^ or on tuberculosis disease screening in migrants from high to low tuberculosis burden settings.^54–57^ This type of evidence makes it difficult to establish whether any increase in tuberculosis disease in migrants compared with non-migrants from the same region is driven by an increase in transmission and infection resulting from changing living conditions and social contact during transit, in camps, and asylum settings; or due to progression to tuberculosis disease due to poor nutrition or poor access to preventive care because of barriers such as language, legal status, low resources, stigma, or a combination of these factors. Although we focus here on tuberculosis infection, it is important to highlight that a combination of several factors described above, including underlying health vulnerabilities, play a role in increasing disease risk throughout the migration and displacement journey.^53^ In the context of human mobility and tuberculosis infection, a review of the impact of conflict on infectious disease found an increase in tuberculosis transmission due to displacement.^58^ A separate review of migrants to low tuberculosis burden settings identified case studies where transmission during transit led to geographically widespread clusters.^59^ Another review comparing transmission in foreign-born and native-born communities,^60^ found that tuberculosis in foreign-born populations did not have a substantial impact on tuberculosis among native populations in Europe. One individual study compared tuberculosis infection prevalence, finding no evidence for a link to living in a disaster area but some evidence for a link to overcrowded living conditions,^61^ whereas a modelling study considering rural–urban migration in China identified the important role of migration in transmission.^62^ Although multiple reviews identified a high risk of tuberculosis infection among migrants across a range of settings, they frequently did not provide direct comparisons with populations of origin to assess changes in transmission risk.^52,63,64^

### Food and water insecurity

#### Evidence on climate change and food and water insecurity

Climate change and food and water insecurity have long been known to be intrinsically linked,^3^ and were the focus of a 2019 special IPCC report.^65^ Approximately 733 million people faced hunger in 2023, equivalent to one in eleven people globally and one in five in Africa, and if current trends continue 582 million will still be chronically undernourished by 2030.^66^ Short-term disruptions to food systems due to climate-driven extreme weather events or other disasters such as earthquakes and conflicts have been shown to have a direct effect on food and water security, as well as nutritional status,^66–71^ with exposure to these acute events potentially leading to lasting consequences.^72^ Meanwhile, longer-term effects of changing temperatures and precipitation on crop yields, grassland quality, and oceans (through warming and acidification) have already been observed to negatively impact agricultural and aquaculture productivity, with substantial future impacts on food security expected.^3,73–75^ Furthermore, a large body of literature exists reviewing the effects of climate change directly on nutritional status and associated health outcomes,^76–78^ and projecting longer-term effects due to changing calorific availability and diets,^79–85^ including because of increased food costs. The focus of many of these studies is on malnutrition in children, including stunting and wasting, or on obesity and overweight in adults. Several studies were also found on the effect of climate-induced food insecurity on birth weight, which are not included here. Importantly, although the effects of climate change on food and water insecurity are likely to affect everyone to some extent, they are likely to disproportionately affect high tuberculosis burden countries with underlying vulnerabilities in their food systems.^3^

#### Evidence on food and water insecurity and tuberculosis disease

Abundant evidence exists linking food and water insecurity to tuberculosis via the pathway of undernutrition. Undernutrition at least doubles the risk of tuberculosis disease, and nearly 1 million cases of tuberculosis globally were estimated to be attributable to undernutrition in 2023,^6^ although this number is likely to be much higher in many high tuberculosis burden countries.^86^ Most studies use BMI, a widely and easily used indicator of nutritional status that has been shown to have a dose–response relationship with tuberculosis incidence in a range of settings and populations.^87,88^ Several other systematic reviews and meta-analyses further support the association between increased tuberculosis disease risk and undernutrition.^89,90^ The reverse has also been shown to be true; interventions addressing food insecurity, such as provision of food baskets, have been shown to reduce tuberculosis disease risk.^91^ In addition, modelling studies projected large reductions in tuberculosis burden if undernutrition is addressed,^92^ and the reverse if undernutrition worsens.^93^ Other important pathways exist by which food and water insecurity affect tuberculosis consequences. Chief among these is an increase in poor tuberculosis treatment outcomes associated with undernutrition,^94–96^ whereas improvements in nutritional status likely improve outcomes.^97^

### Health system disruptions

#### Evidence on climate change and health system disruptions

Around 3·5 billion people live in areas that are highly vulnerable to climate change, with direct consequences for their access to health-care services.^98^ There is a substantial body of evidence linking climate change and health systems disruptions,^3,53,98–100^ showing how climate events compromise health-care infrastructure, disrupt service provision, and strain the health-care workforce. Existing literature on health system disruptions predominantly examined the impact of extreme weather events (particularly flooding and storms) on health-care service delivery, with several reviews emphasising disruptions to chronic disease management.^101^ One review focused specifically on challenges faced by the health-care workforce and how to mitigate these,^102^ whereas another evaluated preparedness of hospitals for disasters.^103^ Further studies from oncology and maternal health highlighted how extreme weather events affect access to health services, particularly for vulnerable populations.^104,105^ These studies provide lessons on changes in health-care utilisation between affected and unaffected communities, and by socioeconomic position. Studies concerning other natural disasters, such as earthquakes and volcanic eruptions, were not included in our review but might still provide relevant insight. Beyond natural disasters, emerging evidence highlights additional climate-related disruptions to health care. For example, a modelling study showed how emergency department visits might change due to increasing temperatures,^106^ whereas another review evaluated the effects of economic recessions (not necessarily climate-induced) on health care.^107^

#### Evidence on health system disruptions and tuberculosis outcomes

Much of the evidence on the effects of health system disruption on tuberculosis focuses on the COVID-19 pandemic, with a strong emphasis on reductions in tuberculosis case notifications, a proxy indicator for the number of people reported to have accessed care. These disruptions alone are estimated to have led to nearly 700 000 excess tuberculosis deaths between 2020 and 2023.^6^ Several reviews collated evidence on the effects of disruptions associated with the pandemic on the tuberculosis care cascade,^108^ with some explicitly considering tuberculosis treatment and outcomes.^6,108^ Meanwhile, modelling studies projected the possible consequences for multiple settings, finding substantial increases in incidence and mortality.^6,109,110^ Due to the nature of the disruptions, most studies combined the effects of disruptions to service delivery and human resources together with disrupted supply chains, with little evidence characterising the effect of disruptions to infrastructure and technologies, energy, or sanitation. There is also little evidence on the effect of disruptions on other outcomes, such as disease severity or catastrophic costs. Outside of the COVID-19 pandemic, two reviews of the effect of conflict on tuberculosis also identified studies from a range of settings, again focused primarily on diagnostic delay and treatment interruption.^53,58^ An earlier modelling study evaluated the effect of an Ebola outbreak, finding a reduction in tuberculosis diagnosis and treatment success.^111^

## Discussion

Through the development of this analytical framework and by undertaking comprehensive literature reviews, we have shown that the effects of climate change on the tuberculosis epidemic are likely to be mediated through multiple pathways and have the potential to be highly consequential. Specific effects will vary by the magnitude of the climate hazard, the vulnerability of communities to their effects (eg, due to differences in underlying tuberculosis determinants), and the capacity of communities to adapt (determined by factors including income, living conditions, and access to health care and social protection). Importantly, people affected by tuberculosis are particularly vulnerable to the effects of climate change because they are already disproportionately more likely to be living in poverty, be undernourished, and have comorbidities, such as HIV. These vulnerabilities hinder their ability to adapt effectively, perpetuating inequality and injustice.

Outlined in the panel are several research gaps evident from our literature reviews and the WHO-convened multistakeholder consultation, which focus specifically on the three climate–health links described previously. Overall, although evidence exists showing the relationship between climate change and each climate–health link (eg, climate change and food and water insecurity); and between each climate–health link and tuberculosis (eg, food and water insecurity and tuberculosis disease), there were no studies directly quantitatively linking climate change and tuberculosis, via any of the described climate–health links or otherwise.

Two other potential pathways, not reviewed in detail here, but that merit further consideration, are air pollution and meteorological factors. Nearly the entire global population breathes air that contains pollutants exceeding the levels recommended by WHO guidelines.^112^ These pollutants, including particulate matter (with a diameter of ≤2·5 μm and ≤10 μm) and sulphur dioxide, are a known cause of major cardiovascular and respiratory diseases, including cancer. Since these pollutants also impair lung defence mechanisms and might modulate the immune response, chronic exposure might increase susceptibility to tuberculosis infection, progression to tuberculosis disease, and risk of adverse tuberculosis outcomes. The association between exposure to household (indoor) air pollution (from burning solid fuels for heating or cooking) and tuberculosis incidence has been established for some time.^113^ For ambient (outdoor) air pollution, a systematic review showed an association with increased tuberculosis incidence,^114^ but not with hospital admission or mortality, and highlighted the low availability and quality of evidence. Further research is therefore required in this area, including research to establish the causal pathways (ie, whether air pollution increases susceptibility to tuberculosis infection, progression to tuberculosis disease, or both). Importantly, ambient air pollution and climate change are strongly interconnected. Many of the causes are the same, principally the burning of fossil fuels and deforestation or agricultural practices, and there are also multiple feedback loops.^115^ Drier and hotter conditions can increase ambient air pollution directly through increased photochemical production and air stagnation events, and indirectly through wildfires.^116^ Many air pollutants (especially black carbon) directly contribute to global warming by absorbing solar radiation and trapping heat in the atmosphere.^117^ Climate change might also increase exposure to household air pollution if extreme heat or weather drives people to spend long periods indoors. These interconnections mean that interventions to address either air pollution or climate change will generally have beneficial effects on the other.

The associations between specific, measurable meteorological factors such as temperature, humidity, and precipitation and tuberculosis have also been the subject of some research, primarily in Asia, and these factors have been proposed as potential indicators that could be used to predict future changes in tuberculosis incidence. Results, however, might be inconsistent. For example, some studies suggest that increases in average temperature are associated with increased tuberculosis incidence,^118,119^ whereas others report that increased temperatures are protective.^120,121^ A systematic review and meta-analysis of meteorological factors and tuberculosis found that tuberculosis risk was positively correlated with precipitation exposures but not average temperature, humidity, air pressure, or sunshine duration.^122^ Another meta-analysis on ecological-level factors and tuberculosis found higher humidity and precipitation were associated with increased tuberculosis incidence, whereas higher wind speed was associated with reduced tuberculosis incidence.^123^ It has also been posited that antimicrobial resistance might independently increase as temperature increases.^124^ Importantly, these associations alone provide little insight into the pathways through which such meteorological factors might impact tuberculosis. Relatedly, seasonal variations in tuberculosis case notifications have been shown in multiple settings,^125^ with explanations proposed ranging from seasonal-related migration (mostly for economic opportunities); to indoor crowding (because of cold weather or extreme heat); and vitamin D deficiency in winter manifesting as a spring peak in notifications. Ultimately, the relationship between meteorological factors and tuberculosis is likely to be highly context-specific, varying by geographic region, local climate patterns, and socioeconomic conditions, and further research is required in this area.

The available evidence shows that efforts to mitigate the effects of climate change on the tuberculosis epidemic should adopt a multisectoral approach that addresses underlying tuberculosis determinants and is also responsive to the unique needs of vulnerable populations, especially migrants and displaced populations. Immediate actions should include meeting core obligations under the right to health by ensuring universal health coverage and establishing a social protection floor for all individuals, complemented by sustained efforts to reduce population vulnerabilities to tuberculosis by pursuing achievement of other key 2030 Sustainable Development Goals (especially related to nutrition and housing) with renewed vigour. Furthermore, adequate investment is urgently needed to build health systems and communities that are resilient to the effects of climate change (especially those caused by extreme weather events in the short-term); and to mitigate adverse economic and non-economic fallouts, especially for vulnerable populations. Using the framework to identify relevant entry points for the three climate–health links discussed here, table 2 outlines some examples of specific measures, which can be mapped to where in the pathway they intervene. However, to support the development of evidence-based policy recommendations in the future, further research is urgently required to develop a prioritised set of feasible, practical, and impactful interventions ultimately aiming to address both the short-term and long-term effects of climate change.

To achieve these objectives, WHO Member States should first recognise the interlinkages between tuberculosis and climate change and ensure The End TB Strategy^16^ is implemented synergistically with other global agreements, especially those focused on preserving our planet. These agreements include The Paris Agreement to limit global temperature rises by reducing greenhouse gas emissions from burning fossil fuels—acknowledging the paradox that continued fossil fuel extraction deepens the very climate crisis that exacerbates the tuberculosis epidemic and poses an existential threat to humanity; the Sendai Framework for Disaster Risk Reduction 2015–2030, and the Global Compacts for Migration and Refugees.^126–130^ National tuberculosis programmes can serve as entry points by monitoring the effects of climate change on tuberculosis to build evidence and support decision making, and foster cooperation with national adaptation policies and institutions. Civil society organisations, particularly those representing tuberculosis-affected communities, play an important role in amplifying the voices of those affected by tuberculosis within climate mitigation and adaptation initiatives, advocating for equitable policies, and mobilising resources. These local efforts should be supported by international institutions and collaborative mechanisms to exchange best practices, share data, strengthen evidence, and propose effective mitigation strategies, drawing insights from other health contexts where applicable. Lastly, the effect of The End TB Strategy on climate should also consider how to mitigate the environmental impacts for all the inputs required to ensure and deliver people-centred prevention, treatment and care, ultimately aiming to encourage tuberculosis programmes to adopt less carbon-intensive measures and move forward carbon-neutral tuberculosis programmes.

For more on **UN Sustainable Development Goals** see https://sdgs.un.org/goals

In conclusion, we have shown how evidence supports the existence of causal links between climate change and tuberculosis, and how further evidence is urgently needed to quantify the extent of its impact on tuberculosis. In the meantime, tuberculosis should be considered as a climate-sensitive disease and be included in climate risk adaptation and mitigation programmes, and climate-resilient funding and response mechanisms. As our work shows, climate change is already hindering progress in the fight against tuberculosis, and only through urgent research and comprehensive action can we address this overlooked intersection and prevent it becoming a barrier to ending the global tuberculosis epidemic.

## Figures and Tables

**Table 1:** Climate–health links prioritised in our Position Paper

	Postulated principle climate factors	Postulated structural and environmental determinant pathway of influence	Postulated social and health determinant pathway of influence	Primary postulated consequence for tuberculosis
Migration and displacement	Changing temperature and rainfall, extreme weather events, sea level rise, and warming oceans	Poverty and inequality, resource crises, humanitarian crises, conflict and violence, ecosystem change, and land degradation and availability	Changing social contact and living conditions	Increased risk of tuberculosis infection, via increased exposure and susceptibility
Food and water insecurity	Changing temperature and rainfall, extreme weather events, sea level rise and warming oceans	Poverty and inequality, resource crises, humanitarian crises, conflict and violence, ecosystem change, land degradation and availability	Changing nutritional status	Increased risk of tuberculosis disease, via increased likelihood of tuberculosis progression
Health system disruptions	Extreme weather events	Humanitarian crises, conflict and violence	Changing access to tuberculosis services	Poor outcomes, via increased vulnerability to tuberculosis and reduced diagnosis and care

**Table 2:** Recommended cross-sectoral intervention entry points

	Examples of interventions	Potential sectors involved in intervention
Migration and displacement	Migration-sensitive tuberculosis screening programmes; infection control and prevention measures; housing programmes; and equitable and affordable access to health care services	Health, migration, and housing or social protection
Food and water insecurity	Sustainable agriculture and water resource management interventions; strengthened food supply chains to ensure accessibility and affordability; social protection programmes targeting vulnerable populations (eg, cash and in-kind transfers); and targeted nutritional interventions to address malnutrition and micronutrient deficiencies	Health, agriculture, and finance or social protection
Health system disruptions	Health system and supply chain strengthening; mobile care and digital health systems ensuring continuity of tuberculosis services and treatment support; and decentralisation of care and training health professionals to manage climate-sensitive health challenges	Health and environment
Planning and coordination in countries most affected by tuberculosis	Integration of tuberculosis prevention and care into national plans of action for climate mitigation and adaptation; and participation of people affected by tuberculosis, particularly those most vulnerable to climate impacts, in policy-making processes related to climate mitigation and adaptation	Health

## Key messages

- Despite the substantial overlap between tuberculosis burden and climate vulnerability globally, the intersection of these two key health challenges remains under-researched and under-represented in climate health policy discussions.
- Through an iterative review process and WHO-convened multistakeholder consultation, we developed a comprehensive analytical framework that maps the potential causal pathways linking climate change to tuberculosis.
- Our review gathered and synthesised the available evidence to support three examples of key pathways through which changing climate factors might have consequences for tuberculosis: (1) migration and displacement; (2) food and water insecurity; and (3) health system disruptions.
- Although we found no direct quantitative evidence on the effects of climate change on tuberculosis, through this process we showed that the effects mediated through these three pathways, among others, have the potential to be highly consequential for tuberculosis.
- Tuberculosis should therefore be considered as a climate-sensitive disease and action should be urgently taken to mitigate the potential effects of climate change on tuberculosis described here, and to develop climate-resilient tuberculosis programmes. To do so, stakeholders should adopt a multisectoral approach that addresses underlying tuberculosis determinants and is responsive to the unique needs of vulnerable populations.
- Alongside these actions, further evidence is urgently needed to quantify the potential effects of climate change on tuberculosis, and to develop interventions to mitigate these effects.
- Civil society organisations representing the voices of tuberculosis-affected communities should be central to these efforts, supported by increased funding from key international institutions, advocacy, and the development of collaborative knowledge sharing platforms to exchange best practices, identify challenges, and propose effective mitigation strategies.

## Box

***Panel:* Examples of research domains and questions to characterise the effects of climate change on tuberculosis, and ways to mitigate, adapt, and build resilience**

**Climate change and tuberculosis**

No evidence exists directly linking climate change to tuberculosis. There is an urgent need to generate such evidence, and to identify prioritised adaptation and mitigation measures for climate-resilient tuberculosis services with a minimal environmental footprint.

### Specific questions

- Are the effects of climate change on tuberculosis likely to be similar in high and low burden settings, and to what extent could climate change impact the global epidemiology and distribution of tuberculosis (eg, could the effects change a country from a low burden to a high burden setting?)
- How do the effects of climate change on tuberculosis vary among different population groups (eg, by gender, or people living in extreme poverty)?
- Which other climate–health links (eg, air quality and extreme heat) might have significant causal effects on tuberculosis, beyond those currently explored here?
- How does the environmental footprint of tuberculosis prevention and care (eg, diagnostics and the treatment process) contribute to climate change, and what sustainable practices can be introduced to minimise this impact?
- What adaptation and mitigation measures (eg, improved housing, social protection, resilient health-care systems) should be prioritised to build climate-resilient tuberculosis services, particularly in resource-constrained settings?

**Climate change, migration and displacement, and tuberculosis infection**

Evidence linking climate change to migration and displacement has focused on population movement in the longer term, particularly due to sea level rise, with less evidence on when this migration and displacement is likely to lead to overcrowding, increases in urbanisation, or poorer living conditions. Evidence linking migration and displacement to tuberculosis infection has focused on tuberculosis disease among migrants in low-burden settings, with little data on how infection risk changes and the effect on refugees, asylum seekers, and internally displaced people.

### Specific questions

- Do existing theoretical and operational frameworks adequately capture the complex, multifaceted effects of climate change on migration, including acute displacement and longer-term population shifts?
- How will overcrowding, poor living conditions, and increasing urbanisation driven by climate change affect tuberculosis transmission risks and health outcomes (including mental health) in displaced populations?
- What are the barriers to health-care access for displaced populations affected by climate change, and how do these barriers influence tuberculosis detection, treatment adherence, and outcomes?
- What social and economic determinants related to climate change and displacement (eg, unemployment, poverty, and food insecurity) most strongly influence tuberculosis risk in displaced populations?
- How does migration type and experience (eg, internal displacement or asylum seeker) alter transmission dynamics, risk of exposure, and health outcomes?
- Can studies quantify the effect of different forms of climate-induced migration and displacement on tuberculosis transmission risk, particularly in high-burden and climate-vulnerable areas?

**Climate change, food and water insecurity, and tuberculosis disease**

Evidence linking climate change to food and water insecurity has focused on children, and adults who are overweight, particularly due to changing food availability and diets, with little data on undernutrition in adults and the effects of extreme weather events and acute nutritional shocks. Evidence linking food and water insecurity to tuberculosis disease has focused on BMI as an indicator, with little data from high tuberculosis burden settings, in populations with a particularly high prevalence of severe underweight, for children and adolescents, or for other nutritional indicators.

### Specific questions

- How will the growing frequency and intensity of extreme weather events and their effects on food and water insecurity subsequently affect susceptibility to tuberculosis infection, disease progression and tuberculosis outcomes, and what are the most effective forms of nutritional support and anthropometric markers to deploy in these settings, both in the short term and long term?
- How does the relationship between BMI and tuberculosis incidence vary across different populations, including children and older people and those with comorbidities, such as HIV infection, alcohol use disorders, or end-stage renal disease; and does the type of weight loss (acute *vs* chronic) or BMI distribution in a setting influence tuberculosis risk, even in the absence of malnutrition?
- Can additional nutritional indicators—such as haemoglobin levels, body composition (via bioelectrical impedance analysis), point-of-care micronutrient testing, and measures of dietary diversity and quality—offer more precise evidence of vulnerability to tuberculosis disease, beyond BMI alone, and what are the most appropriate nutritional metrics for different contexts?
- Can models quantify the added impact of increased food and water insecurity driven by climate change on people vulnerable to tuberculosis?

**Climate change, health system disruptions, and tuberculosis outcomes**

Evidence linking climate change to health system disruptions has focused on extreme weather events and service delivery or access, with little evidence on gradual disturbances from repeated shocks or climate-driven economic slowdowns, or the effect on supply chain disruptions, infrastructure, energy, and sanitation. Evidence linking health system disruptions to tuberculosis outcomes has focused on the COVID-19 pandemic and case notification, with little evidence from other forms of disruptions, or for a wider range of health and socioeconomic outcomes.

### Specific questions

- How will factors beyond extreme weather events, such as prolonged economic crises or extreme heat, contribute to health system disruptions, and how will these affect tuberculosis service delivery?
- In what ways will climate change impact essential health service functions (eg, supply chains, infrastructure, energy availability, health workforce, and sanitation) critical to effective tuberculosis response?
- What are the long-term consequences of health system disruptions caused by climate change on tuberculosis-related health outcomes, including the risks of chronic lung damage, the acquisition of drug-resistant tuberculosis strains, and financial burden on affected populations?
- Can experience from previous disruptions be used to quantify the effects of acute climate events (eg, floods or droughts) on tuberculosis outcomes as a result of disrupted service delivery, including disruptions to tuberculosis diagnostics, supply chains, and treatment delivery?

## Search strategy and selection criteria

Our search strategy combined searching abstracts and titles for key words with MeSH terms. For example, to generate evidence linking climate change to changing tuberculosis infection because of migration and displacement, one search combined the concepts: climate change with migration and displacement, and study type; and a second combined migration and displacement with tuberculosis, infection, and study type (exact search terms provided in the appendix pp ^1–3^). Although our search was limited to MEDLINE, potentially limiting the comprehensiveness of our review, we further increased our body of evidence by checking references of included articles, citation forward searching (checking for articles that cited included articles), and consulting key experts to obtain information on recent or ongoing studies not captured in our searches. Given the broad scope, our search strategy was refined iteratively, including by identifying MeSH terms and key words for relevant articles and adding them to our search terms if not already included. We screened titles and abstracts returned by the searches and accessed full-text versions for those potentially eligible for inclusion, including studies in human populations published from Jan 1, 2000, to June 20, 2024, in English. For pragmatic reasons, only evidence from modelling studies or pre-existing reviews (including systematic reviews, meta-analyses, scoping reviews, and narrative reviews) was included. Individual studies, case reports, and perspectives were considered only when no reviews or modelling studies could be found, or where they provided a novel perspective. The results of the searches are summarised in the appendix (p 4), and additional relevant evidence not cited here is also included (pp 5–17). Key data from included articles were extracted into a standardised form. We then conducted a narrative synthesis to summarise key findings relevant to each topic and used this synthesised evidence to adjust and refine the analytical framework towards its final state, including highlighting key areas critically lacking in evidence. This framework refinement was an iterative process, further informed by discussion with other experts and key stakeholders at the WHO multistakeholder consultation.
